# Artificial intelligence based data curation: enabling a patient-centric European health data space

**DOI:** 10.3389/fmed.2024.1365501

**Published:** 2024-05-15

**Authors:** Isabelle de Zegher, Kerli Norak, Dominik Steiger, Heimo Müller, Dipak Kalra, Bart Scheenstra, Isabella Cina, Stefan Schulz, Kanimozhi Uma, Petros Kalendralis, Eno-Martin Lotman, Martin Benedikt, Michel Dumontier, Remzi Celebi

**Affiliations:** ^1^B!loba, Tervuren, Belgium; ^2^North Estonia Medical Centre, Tallinn, Estonia; ^3^Department of Health Technologies, Tallinn University of Technology, Tallinn, Estonia; ^4^MIDATA Genossenschaft, Zürich, Switzerland; ^5^Diagnostics and Research Institute of Pathology, Medical University Graz, Graz, Austria; ^6^The European Institute for Innovation Through Health Data, Ghent, Belgium; ^7^Department of Cardiothoracic Surgery, Cardiovascular Research Institute Maastricht, Maastricht University Medical Centre, Maastricht, Netherlands; ^8^European Heart Network, Bruxelles, Belgium; ^9^Averbis GmbH, Freiburg, Germany; ^10^Institute for Medical Informatics, Statistics and Documentation, Medical University Graz, Graz, Austria; ^11^Faculty of Engineering Science, Department of Computer Science (HCI), Leuven, Belgium; ^12^Department of Radiation Oncology (Maastro), GROW School for Oncology and Reproduction, Maastricht University Medical Centre, Maastricht, Netherlands; ^13^Department of Internal Medicine, Division of Cardiology, Medical University of Graz, Graz, Austria; ^14^Department of Advanced Computing Sciences, Institute of Data Science, Maastricht University, Maastricht, Netherlands

**Keywords:** AI-based data curation, personal health knowledge graph, ontology, catalogue of data sources, EHDS, data intermediary, patient-centricity

## Abstract

The emerging European Health Data Space (EHDS) Regulation opens new prospects for large-scale sharing and re-use of health data. Yet, the proposed regulation suffers from two important limitations: it is designed to benefit the whole population with limited consideration for individuals, and the generation of secondary datasets from heterogeneous, unlinked patient data will remain burdensome. AIDAVA, a Horizon Europe project that started in September 2022, proposes to address both shortcomings by providing patients with an AI-based virtual assistant that maximises automation in the integration and transformation of their health data into an interoperable, longitudinal health record. This personal record can then be used to inform patient-related decisions at the point of care, whether this is the usual point of care or a possible cross-border point of care. The personal record can also be used to generate population datasets for research and policymaking. The proposed solution will enable a much-needed paradigm shift in health data management, implementing a ‘curate once at patient level, use many times’ approach, primarily for the benefit of patients and their care providers, but also for more efficient generation of high-quality secondary datasets. After 15 months, the project shows promising preliminary results in achieving automation in the integration and transformation of heterogeneous data of each individual patient, once the content of the data sources managed by the data holders has been formally described. Additionally, the conceptualization phase of the project identified a set of recommendations for the development of a patient-centric EHDS, significantly facilitating the generation of data for secondary use.

## 1 Introduction

The European Health Data Space (EHDS) draft Regulation published in May 2022 (1) is a ground-breaking initiative which aims to unlock the full potential of health data by facilitating their secure exchange and reuse across the European Union. While the EHDS opens unprecedented opportunities for the management and exploitation of health data, the proposed implementation suffers from two important limitations.

Firstly, the EHDS is designed to benefit the whole population with limited consideration for individuals: it regulates how to manage data for analysis and decision-making across the population, while its usefulness for individual patients in day-to-day care is limited. The main benefit for individual patients, will be the availability of six categories of personal health data—including patient summary, laboratory results, prescribing and dispensing information, imaging reports and discharge summaries—in an interoperable and standardised digital format; this will enable smooth exchange of critical personal health information between healthcare providers across Europe and beyond, primarily for unplanned care needs. Patients will also be able to access their data through National Contact Points for Digital Health (NCPDH); these public health organisations have no direct contact with patients and therefore have little opportunity to establish a relationship of trust at an individual level. While the EHDS will bring benefits to patients, there is a missed opportunity for individuals to actively participate in managing, completing, and improving the quality of their own medical records, which are made of disparate data sources with inconsistencies, gaps and limited interoperability and reuse.

Secondly, the generation of secondary datasets in EHDS will continue to require recurrent curation of potentially identical patient data and provide sub-optimal datasets. Health Data Access Bodies (HDABs), which are also public health organisations, will be granted permission—with opt out possibility for the patients—to process patient data for secondary use by authorities and researchers. As source patient data will remain heterogeneous, there is a risk that the HDABs will process the same data several times for different purposes. Furthermore, as patient data cannot be linked^1^ without subjects’ consent or in crisis situations, the resulting population datasets can only provide partial views of patients, with sub-optimal data quality.

AIDAVA (2)—a 4-year Horizon Europe project launched in September 2022 with 14 partners, under grant agreement 101057062—proposes a new paradigm in health data management by giving patients greater control and agency (3) over their personal health data through an intelligent virtual assistant (VA). The AIDAVA solution will first help patients to integrate their data collected by hospitals, general practitioners, patient-reported outcome management systems (4), and medical devices. It will then use multiple curation technologies to semi-automatically transform this data into a formal, interoperable representation based on knowledge graph technology (5), called the Personal Health Knowledge Graph (PHKG) (6). Each PHKG is constrained by the AIDAVA reference ontology to ensure interoperability and maximise reuse; the reference ontology (7) will built on ontology frameworks from standards in use in the European Electronic Health Record Exchange Format (EEHRxF) (8)—including HL7 FHIR, SNOMED, LOINC standards—and in clinical research, such as CDISC and OMOP.

During the curation and publishing processes, the VA will request feedback from the individual when full automation cannot be achieved; for complex questions, the VA will request the contribution of an expert data curator. To increase the understanding of the question and the quality of the response, the VA will provide contextual information using metadata regarding the data sources and their transformations and considering the level of health and digital literacy of the patient.

AIDAVA has the potential to implement the ‘curate once at patient level, use many times’ principle for the benefit of the patients and their care providers. From the interoperable personal longitudinal health record derived from multiple heterogeneous data sources, AIDAVA will be able to generate, on request, the six priority personal health data in EEHRxF format, as well as data extracts complying with national specifications and future versions of EEHRxF. In addition, the availability of multiple, interoperable PHKGs accelerates—with permit or dynamic patient consent—the smooth generation of secondary use datasets, with superior quality because data are linked at the individual level within each PHKG.

This paper first presents the perceived limitations of the EHDS regulation and introduces the potential of data intermediation services described in the Data Governance Act (9) to manage personal health data. It then describes the ongoing research topics developed within the AIDAVA project. Finally, it proposes preliminary recommendations for an innovative digital health infrastructure that promotes seamless data integration, interoperability, and data quality for individual health data, thereby improving patient care, research capabilities and the efficiency of the healthcare system. The authors suggest integrating these preliminary recommendations into the implementing acts currently being drawn up for the deployment of the EHDS.

## 2 Materials and methods

### 2.1 Review of EHDS

#### 2.1.1 EHDS is authority and population centric rather than patient-centric

At the heart of health data management are the data holders who collect personal data, including clinical data, social determinants of health and clinical research data^2^. The GDPR data portability right (10) enables individuals to move, copy, or transfer their personal data across data holders; the emerging Data Act (11) will further regulate the portability of data from Internet of Things and medical devices data holders in particular.

**Figure 1:**
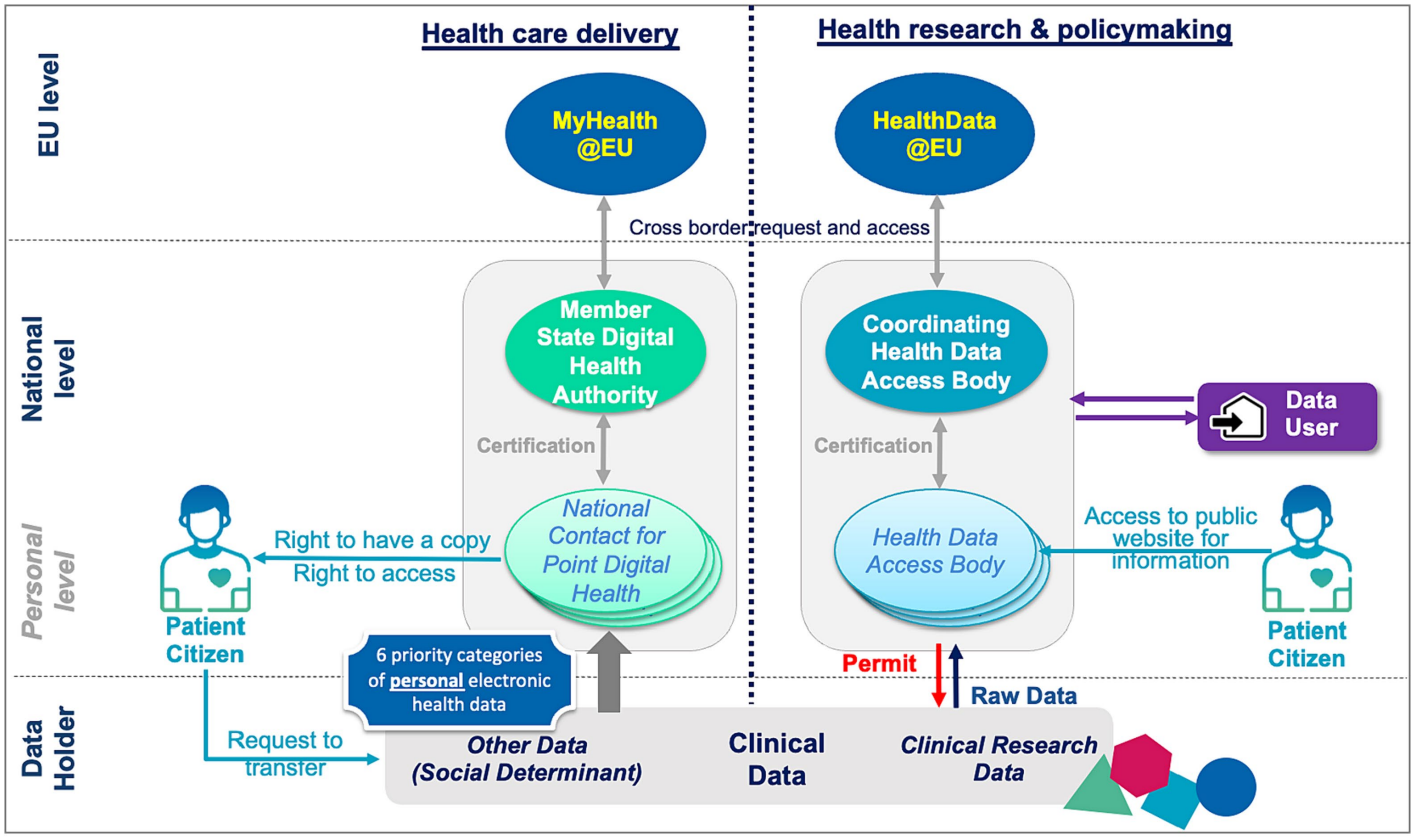
Organisations and main information flows within proposed EHDS regulation.

The EHDS proposes the creation of four different types of organisations within Member States and two at European level, across health care delivery and research (Figure 1).

Organisations on the health care delivery side include: (i) *National Contact Points for Digital Health (NCPDH)* which act as gateway for European citizens to access their data, pooled from data holders, (ii) a *Member State Digital Health Authority* which is responsible for enforcing the lawful use of data in health care delivery, certifying and supervising NCDPHs and cooperating with other Digital Health Authorities and the Commission, and (iii) *MyHealth@EU* which supports the infrastructure for cross-border management of health care delivery data.

Patients have the right to request data holders to transfer their data to a NCDPH, and to access their data from this NCDPH; patients can also request a free copy of their data, in the state they are at the NCDPH. Finally, patients will benefit from six priority categories of identifiable data in a standardised digital format they can share with healthcare providers throughout Europe to ensure safer unplanned care when travelling.

Organisations on the research and policymaking side include: (i) *Health Data Access Bodies (HDAB)* which are responsible for processing health data for secondary use on the basis of the conditions specified in the regulation, (ii) a *Coordinating Health Data Access Body* which enables the cross-border secondary use of electronic health data under the responsibility of each Member State, in cooperation with other coordinating bodies and the Commission and (iii) *HealthData@EU* which supports the infrastructure for cross-border use of research and policymaking data.

Data users, defined as any natural or legal person who have lawful access to personal or non-personal electronic health data for secondary use, may submit a data access application to a HDAB for any purpose identified in the regulation. Patients who wish to understand how their personal health data are used, can access a public website where the HDABs register the permits they have been granted.

Except for data holders and data users, all organisations mentioned above are public organisations or research infrastructure established as a European Research Infrastructure Consortium, funded per Member State and/or the European Commission. Private organisations are not mentioned, while they can bring a wealth of expertise and know-how in processing health data and can stimulate a true data economy benefiting the patients. This is particularly the case for emerging data intermediaries, regulated by the Data Governance Act; they could provide data intermediation services to patients enabling them to exercise their GDPR right to correct errors, and to curate and improve the quality of their health data before it is sent to the NCDPH. Article 13.2. mentions that Clinical Patient Management System may become authorised participants to MyHealth@EU; there is however no further details.

In addition, the EHDS tends to create a barrier between health care delivery, and health research and policymaking. More specifically, Section 2 seems to consider that primary use of data is synonymous with health care delivery (including home care, primary care, secondary care, and tertiary care), while Section 4 considers that secondary use is synonymous with health research & policymaking, where population datasets are generated from data extracted from individuals’ clinical data and other, personal and non-personal, data.

**Figure 2:**
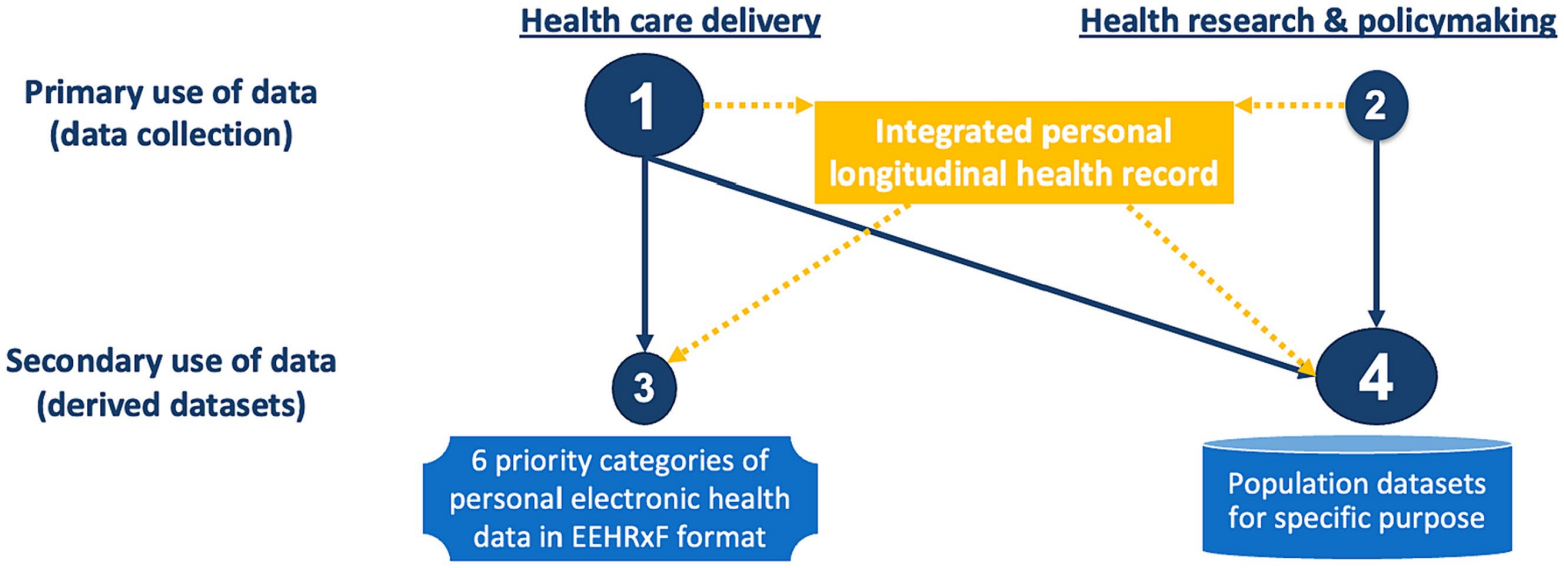
The continuum of health data across delivery and research. Orange indicates suggested data and data flows. Missing in the regulation.

This is confusing against the concept of primary use of data, i.e., data collected for a specific purpose, and secondary use of data, i.e., reuse of existing data for a different purpose. As displayed in Figure 2, data collection is most often taking place in health care delivery, but it is also happening in research (e.g., interventional clinical trials, adverse events, and clinical registries), and policymaking (e.g., public health surveys). For a true patient-centric EHDS, all personal data related to a patient should be first integrated into their personal longitudinal health record, from which different types of data can be derived for health care delivery as well as research and policymaking.

#### 2.1.2 EHDS does not solve the burden of recurrent curation

Health data are heterogeneous because many legacy systems are still up and running—and may remain so for a long time—with large portions of unstructured variables and narrative text. Additionally, multimodal data (medical imaging, genomic data, EHRs, wearable…) require different types of representation and technologies. Lastly, data standards for clinical care and clinical research have different requirements: for instance, HL7 FHIR is structured vertically, gathering all data for a single patient encounter, while CDISC SDTM and ADaM in clinical research organise the same parameter horizontally, for multiple patients.

**Figure 3:**
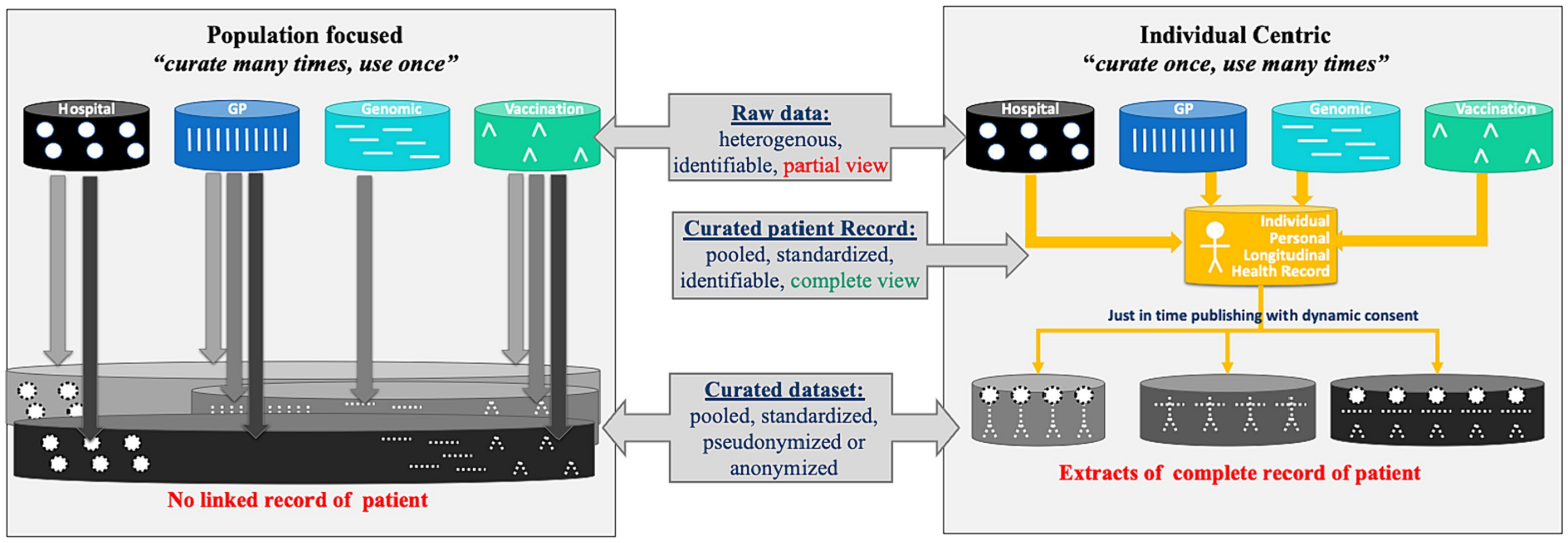
Population focused data curation, vs. individual centric data curation.

For the foreseeable future, health data curation will continue to be necessary. As displayed in Figure 3, the current ‘population-based’ model relies on expert data stewards extracting pseudonymised data from data sources for a specific purpose and transforming this data into the format required for the analysis. As the GDPR regulation does not permit linkage of personal data without a legal basis or personal consent, and as consent of each relevant individual is difficult to obtain with the existing infrastructure, health data are most often not linked, and the curated data provides only a partial view of the patients. In addition, the number of subjects is often different from one data source to another. Finally, as each secondary use may require slightly different datasets, one individual’s data may be curated several times, resulting in massive and unnecessary duplication of effort.

Secondary use in the EHDS follows this model; indeed, the raw data available within the data holders is neither standardised nor linked. Furthermore, while the EHDS regulation introduces basic requirements for quality of the source data, there is no provision for data quality labelling in secondary datasets.

Another concern is that EHDS may become a contributor to additional Greenhouse Gas Emissions (GHGE). After painstakingly generating secondary datasets, HDABs will not be inclined to delete them even though the likelihood of reuse is low; in addition, they might be forced to keep these datasets for liability purposes. Data centres accounted for more than 2.5% of GHGE in 2022, and are targeted to rise to 14% by 2040; 30% of the world’s data volume is generated in the health sector (12) and is expected to rise to 36% by 2025. More than 90% of the data stored in data centres are not used more than once (13).

In a patient-centric EHDS, it is possible to shift the paradigm towards ‘individual centric curation’. The patient, their delegate and/or an agreed expert data curator, curates all their health data, linked across data sources, with the help of an intelligent virtual assistant (VA); the VA orchestrates multiple tools to maximise automation in data curation and quality checks, and involves the patient only when clarifications are required. The result is a personal longitudinal health record, which could be used by attending physicians in the interest of the patient, and by the patient for shared decision-making, second opinion seeking or cross-border care. In addition, if these longitudinal patient records are interoperable, they can be used to generate just-in-time secondary datasets with a quality label derived from the patient records they are extracted from. These datasets could also include metadata—including the programme used to generate them—supporting re-generation of the dataset if needed.

The automated generation of an interoperable, reusable, high-quality, personal longitudinal health record, with and by the patient, is the main objective of the AIDAVA project presented in this paper.

### 2.2 Data intermediaries and data governance act

The Data Governance Act introduced in November 2019 is in force from September 2023, with a transition period of 2 years. It establishes the foundation for data intermediation services, through public and private data intermediary organisations, for public and business data. It also regulates data altruism, i.e., data voluntarily made available to data altruism organisations for the common good, to reduce the cost of collecting consent and facilitate data portability throughout Europe. The Data Governance Act applies to all sectors, including health.

Although data intermediation services were not initially intended to regulate the sharing of personal data, they can naturally be extended to personal data intermediaries, with a set of structured services as described in the MyData Operators Framework (14), following different business models (15). The draft EHDS regulation only mentions data altruism, which benefits authorities but brings limited value to citizens and patients. As advocated by the AIDAVA project, to be patient-centric, EHDS should include personal health data intermediation services, through dedicated and certified organisations called Health Data Intermediaries. These organisations can serve as trusted partners for patients to control the integration, curation and quality of their data, and to manage their preferences for sharing their data before it is reused in care delivery, research and policymaking.

This approach is the cornerstone of a patient-centric EHDS. If it is easy—hopefully seamless—for patients to manage and curate their data while benefiting from an integrated harmonised health record, they will be more likely to engage in managing their health data and in sharing them for the benefit of the population, and ultimately in managing their personal health.

### 2.3 Introduction to the AIDAVA project

**Figure 4:**
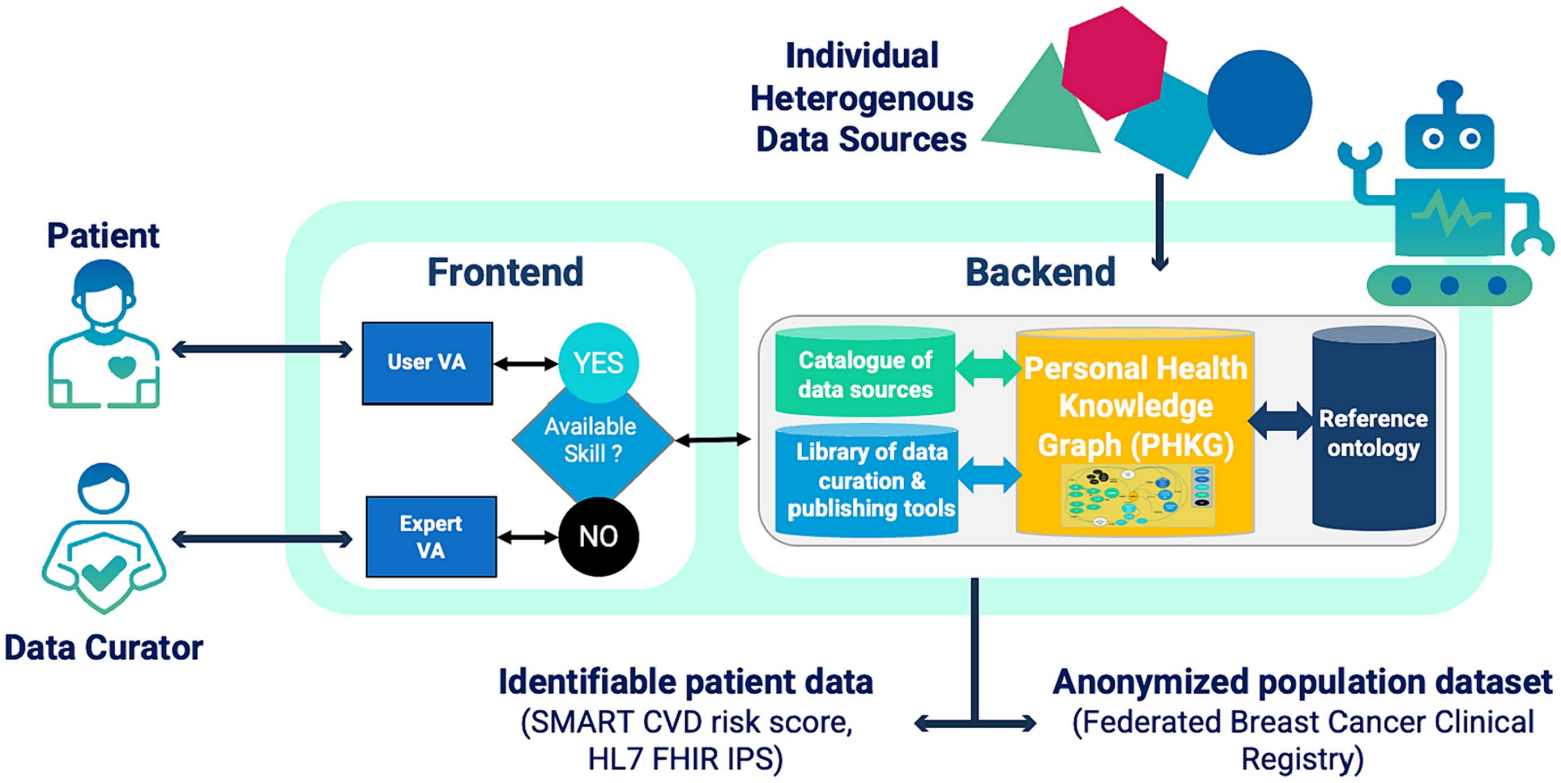
Overview of the AIDAVA Virtual Assistant.

The main objective of the AIDAVA Horizon Europe project is to deliver and test a prototype intelligent virtual assistant (VA) that will assist patients in curating their heterogeneous, multimodal, personal health data into an interoperable Personal Health Knowledge Graph (PHKG). Individuals’ PHKGs can then be transformed into multiple formats for reuse and sharing (16) (Figure 4).

The VA is intended to be used by the patient, or their delegate, and a specialised data curator assisting the patient. The solution aims to maximise automation of the curation process by orchestrating the execution of complementary AI-based curation tools according to the data interoperability issue found in a data source. When automated curation is not achievable, the VA initiates a dialogue with the patient, based on their preferences and skill levels, and provides explanations of the question at hand. Questions which cannot be answered by the patient are addressed to the supporting data curator.

To demonstrate the ‘curate once, use many times’ principle, the AIDAVA VA will generate two types of results from the patient’s PHKG: (i) fully identifiable data extracted from a single patient’s PHKG, in the form of the patient’s cardiovascular risk score and International Patient Summary (IPS) in HL7 FHIR format^3^, and (ii) anonymized population datasets extracted from multiple PHKGs to form an interoperable, site-specific breast cancer clinical registry that can be federated with other sites.

The project builds on four pillars described in the next section: (i) a structured and repeatable curation process enabling automation by orchestrating the execution of multiple data curation and quality enhancement tools, (ii) a reference ontology as a universal data sharing standard (17), supporting European standards and ensuring interoperability of the resulting PHKGs, (iii) a machine-human interaction module generating personalised explanations of the problem to be solved, and (iv) patient engagement through a trusted health data intermediary.

There will be two generations of the AIDAVA VA prototype. Generation I will include the prototype framework consisting of a Chatbot-like platform as the front-end, and orchestration of a library of data curation & publishing tools at the backend. These tools will preferably be off-the-shelf and open source. Generation II will build on the previous generation: the front end will be extended with an explainability module to increase usability for users less experienced in curating data and in medical content; several curation and publishing tools will be updated with tools developed in the project, including multi-lingual AI based Natural Language Processing (NLP) solutions. Each generation of the prototype will be tested in three clinical sites (Universiteit Maastricht in the Netherlands, Sihtasutus Pohja-Eesti Regionaalhaigla in Estonia, Medizinische Universität Graz in Austria) with the support of two data intermediary organisations (MIDATA Genossenschaft for Estonia and Austria, Digi.me Limited for The Netherlands). As AIDAVA is a prototype, it is not subject to the Medical Device Regulation (18). However, as the prototype will be tested with site patients, the evaluation will follow a strictly defined process, documented in a research protocol which must be approved by the local ethical committees.

To ensure a true patient-centred approach, the project is supported by eight patient ‘consultants’ from the European Patient Centre Coalition for Breast Cancer and the European Heart Network for Cardiovascular Diseases. These patient consultants are actively involved at regular, well-defined times for a total of 42 person days per patient throughout the project. They ensure the project stays focused on what is important for patients.

## 3 Results (interim)

The AIDAVA project has been active for 15 months during which the consortium detailed the use cases, the requirements, and the solution architecture, and initiated the development. In parallel, the consortium developed the study research protocol needed for evaluation, as well as the data sharing agreement with data transfer technical specifications for each contributing site. This section describes the interim results.

### 3.1 Automated curation

The first objective of AIDAVA is to automate as far as possible the curation process, transforming heterogeneous health data into a single, harmonised Personal Health Knowledge Graph (PHKG). The curation process involves resolving interoperability issues across these heterogeneous data. Although interoperability has been widely described in different frameworks (19, 20) and publications (21), automation requires a holistic solution based on a precise classification.

We analysed in more detail the issues that hinder data interoperability, differentiating between issues within individual data sources (single-source data interoperability) and issues when integrating data from multiple sources (cross-sources data interoperability). We identified 11 data interoperability issues based on the analysis of the data sources selected in the project, and literature reviews. The single-source issues comprise digitalisation of paper documents, extraction of structured data from free text, format alignment, transformation of semi-structured and structured data, reference data management, terminology alignment, medical coding, and imaging readability. The cross-sources issues include entity deduplication, semantic inconsistencies, and semantic incompleteness.

For each data interoperability issue, we defined a workflow maximising automation in the transformation of the data into a semantically sound knowledge graph. Each workflow uses one or more curation tools supporting resolution of the issues; candidate tools that could be reused or improved were identified. As new, improved tools [e.g., NLP tools based on Large Language Models (22), data wrangling (23) and AI medical coding (24…)] are emerging, they will be replacing older tools. We also specified within the workflows the need for human intervention to resolve issues; approaches to obtain answers from patients, or their supporting curator, are further described in Section 3.3. Finally, we defined a high-level orchestration workflow to deal with multiple data interoperability issues within one data source.

For semantic inconsistencies and semantic incompleteness, the workflow includes data quality rules with triggers for human intervention in case of errors. Data quality rules represent common sense knowledge (e.g., the discharge time in an hospital must happen after the admission time), physio-pathological knowledge (e.g., a breast tumour must include a laterality) and clinical care pathway information (e.g., diabetes type 1 requires an insulin related treatment). Data quality rules also provide a labelling mechanism to assess the reliability of the curated PHKG. For example, curation through a validated and deterministic tool would score higher than curation through an emerging AI tool. Similarly, human input from staff with high health literacy would have a higher score than input provided by a patient with a limited health literacy. A data quality checker is being implemented, together with a governance process to include new rules or remove existing ones. Governance is particularly important, as knowledge encompassed in data quality rules applies to the whole medicine and requires knowledge elicitation, out of scope of the project.

To maximise automation, we needed a preliminary step called ‘*data source onboarding’*, in which metadata on each data source is defined and stored in a dedicated catalogue. This catalogue of data sources includes FAIR metadata enriched with (i) information on the structure and content of the data, such as data type, value restriction and value set, (ii) provenance information related to creation, modification and validation of the source information (25, 26), and (iii) semantic mapping with concepts defined in the reference ontology. Metadata on data sources is collected once, in each data holder organisation; it is used each time the system ingests and curates the data of a specific patient. The AIDAVA catalogue of data sources is being developed on top of DCAT-3 (27); it will be extended with the Data Source Description Vocabulary (28) supporting semantic annotation and the RDF Mapping Language (29) for mapping.

### 3.2 Personal health knowledge graph: interoperability and reuse through reference ontology

A Personal Health Knowledge Graph (PHKG) is a dynamic, semantic representation, which can harmonise and link multimodal, heterogeneous data during the data curation process. Such a PHKG is ideally positioned to capture the semantics of a data source, independently of its structure; it can also support data integration, data quality enrichment and correction, based on the context. Although a PHKG is personal and contextual, it will be interoperable due to being an instance of the reference ontology. As such, the PHKG constitutes a high-quality, FAIR, longitudinal health record, growing continuously as new data is being ingested. During the data publishing process, the data contained in the PHKG can be made available for multiple purposes in the appropriate format.

Achievement of interoperability is constrained by the availability of a commonly agreed and used reference ontology. AIDAVA identified strategic and content requirements for such an ontology. The strategic requirements include (i) support the European Electronic Health Record Exchange Format (EEHRxF), (ii) maximise potential for reuse of the PHKGs across a large range of use cases, beyond the ones identified in the project, (iii) ensure alignment with standards in place to minimise the need of mapping from and to these different standards, while maximising reusability of the PHKG during and after the project, (iv) support maintainability and extensibility during the project as well as beyond the project, and (v) enable implementation and update of constraints supporting data quality.

In terms of content, the ontology will include (i) standards such as SNOMED CT, LOINC, HL7 FHIR General-Purpose Data Types, and HL7 FHIR resource related to the International Patient Summary (30), (ii) concepts that support mapping and transformation with entities and relationships included in the data sources^4^, (iii) predefined mapping supporting transformation to HL7 FHIR IPS and other data exchange messages required by EHDS, and (iv) data quality checks implemented through SHACL rules (31).

We are currently assessing how to use the Swiss Personal Health Network framework (SPHN (32)) as the basic schema of the AIDAVA reference ontology; preliminary results demonstrate that an ontological foundational layer will be needed to support extension of the SPHN schema.

### 3.3 Human-in-the-loop and the value of explainability

AIDAVA emphasises the importance of making the use of AI solutions transparent, and inherently human-inclusive, with interface components adapted to different types of users. Following user-centred design, the project identified eight user personas across different user groups. Personas are fictional characters who represent the similarities of target user groups and play a pivotal role in ensuring that human-AI interaction is tailored to individual needs, promoting more meaningful engagement. To turn the fictional persona into a tangible, realistic character, and to make it easier for system designers to empathise with the user represented by a persona, the latter is visualised in a one-page layout, called a ‘persona canvas’, which includes narrative text about the persona’s interests, preferences, behaviour patterns and attitudes. Within AIDAVA, personas also serve as the foundation of the explainability and feedback with patients, based on their level of digital and health literacy assessed when setting up the user account and stored in their user profile (33).

Most people are not prepared for unmediated interactions with a digital solution that aims to curate their personal health data. To increase acceptance and democratise personal data curation, AIDAVA aims to maximise automation to minimise user intervention. When automation is not possible, and humans must be brought in the loop, AIDAVA will first decide if the question must be raised to the patient or to the supporting data curator, based on the health and digital literacy levels of the patient. In a second step, the system will raise the questions and generate context-based explanations using the type of issue identified in the workflow, the expected human intervention to solve the issue, the level of digital and health literacy of the target user, and the context of the issue to be solved. Context encompasses all aspects of the data’s origin, including information on its creation in the data sources—stored as FAIR metadata available in the catalogue of data sources referred above—and the transformation steps that took place during the curation process. Generation of narrative explanations will be based on canned text translated in each user language for Generation I of the AIDAVA prototype. For Generation II, we are exploring the use of multi-lingual Large Language Models.

### 3.4 Health data intermediaries

AIDAVA is proposing to provide data intermediation services to patients through organisations called Health Data Intermediaries (HDI), introduced in Section 2.2. These emerging organisations, regulated by the Data Governance Act, are expected to provide three services. First, they should operate as a ‘personal health data hub’ integrating multimodal data, sourced directly from the patient (wearables, lifestyle data, etc.) or from healthcare providers who treated the patient (34). Second, HDIs should enable dynamic management of consent for data sharing, via a digital app. For example, the patient could specify for which purpose their data (i) can always be shared, without their consent (e.g., public health purpose), (ii) can be shared with their consent when the purpose is clarified (e.g., clinical trials) and (iii) should never be shared (e.g., marketing and commercial research). Finally, HDIs should support the improvement and labelling of the quality of the patients’ health data; this would increase the value of reuse of this data, firstly for the patient and their treating physician and secondly for research and policymaking.

NCDPHs described above could become health data intermediaries powered by AIDAVA-like virtual assistants. Alternatively, HDIs could serve as smaller data intermediation organisations that assist patients in integrating and curating their data, before it is transferred at NCDPH level.

Within AIDAVA, we are working with two emerging data intermediaries—MIDATA, and DIGI.me—already active in health with the first two functions (personal health data hub and consent management). We are assessing the opportunity of adding the quality enhancement and labelling tools.

## 4 Discussion

Although we are still in the conceptualisation phase and have yet to evaluate the prototype in real-life situations with patients, some preliminary conclusions can already be drawn and lead to recommendations for a patient-centred implementation of the EHDS; these recommendations will have to be confirmed as the project develops.

### 4.1 AIDAVA-like solutions are needed for the benefit of the patients and their treating physicians

In the EHDS, as in many research projects, the focus is on improving the production and quality of targeted secondary datasets for research and policymaking, following the ‘population curation’ approach described in Figure 3. We argue that a paradigm shift is needed towards ‘individual curation’, improving the management of patient data at the point of care, and supporting smoother extraction of secondary datasets from these high-quality, interoperable patient records. This is particularly relevant for patients with complex conditions, as their data accumulates across multiple stakeholders and episodes of care over time.

A patient-centric approach makes it possible to prioritise patients’ interests and needs for day-to-day care by providing a complete medical record that is easily accessible by attending physicians, thereby reducing their daily workload, which in turn decreases the risk of burnout (35). Regarding the use of secondary data, it has been shown that patients are generally in favour of sharing their health data for the common good (36) provided there is transparency, accountability and no data privacy risk. Therefore, the secondary use of patient data for public health purposes could be the default, with the possibility for patients to opt out.

Today, it is extremely difficult for patients to manage and integrate data across different systems, and thus provide a holistic view of their health status. It is equally difficult for them to share information with their treating healthcare providers. Additionally, there is currently no easy way to opt out of sharing their data whenever used for lawfully agreed public health purposes.

AIDAVA-like solutions, in which all data sources have been onboarded as described previously, would enable the patients to control all their health data, to download them from various data sources, curate them into their PHKG and provide consent for sharing. Through AIDAVA-like solutions, patients, or their delegate, would ensure that their data is integrated and of the highest quality, facilitating medical decision-making. In addition, the availability of interoperable PHKG would facilitate the creation of high-quality datasets for research and policy development.

**Recommendation 1**. EHDS, as a patient-centric solution seeking to bring benefits to European citizens, should first consider the benefits to each individual patient; and more specifically seek digital solutions that enable every European citizen to maintain an interoperable, high-quality ***personal longitudinal health record***, usable at the point of care and allowing the smooth generation of secondary datasets for lawful public health purposes.

### 4.2 The major problem in data interoperability and reuse of health data is the lack of documentation on data source

The classical concern about accessing personal identifiable data is local data privacy and protection constraints as well as Ethical Committees’ approvals. This is a time-consuming process, though generally well described, clear and manageable. We realised, however, that access to detailed descriptions of health data available within an organisation was unexpectedly difficult; this includes data schema—technical description of each data element collected within the different subsystems of the organisation—data lineage and data quality labels. Without such documentation, automation as proposed in AIDAVA is not possible and the ‘curate many times, use once’ model will remain the standard, burdensome practise.

Other European projects were faced with the same issue; see for instance ‘Deliverable 2.1. Overview of data sources and plan to access available data sources’ in Precise4Q (37). Documentation of an extract of the patient data in standardised format—related to the six priority categories of personal data to be exchanged per EHDS—starts to be available in several European countries [e.g., in (38, 39)]. This is not enough; all data sources must be documented. To our knowledge, the only country where detailed description of all collected health data is available is Finland (40) as this is mandated by law since 2013.

Secondary datasets also suffer from the same lack of documentation of data elements, which hampers their reuse. Article 37 (i) of EHDS requires each member state to maintain a catalogue of national datasets with details of the source, scope, main characteristics of the population included in the dataset and conditions of access and use. There are no requirements however to provide a detailed description of the data elements included in the catalogue. The EHDS2 pilot project highlighted the importance^5^ of including such information in national catalogues to facilitate interoperability and reuse (I and R in the FAIR principles) of the datasets generated across Member States.

In AIDAVA, we worked for several months with the clinical evaluation sites, to identify and collect the schema of data elements collected at the point of care, supporting automation and explainability in case of human intervention. This information will be stored in the AIDAVA catalogue of data sources, based on existing standards as described in Section 3.1.

**Recommendation 2**. In alignment with Article 23.3 (a) and (b) of the EHDS regulation, implement catalogues of data sources with detailed description of each data element collected by relevant data holders.

- Develop a standard describing the content of a catalogue of data sources; this standard should build on existing standards such as DCAT and Data Source Description Vocabulary.
- Provide an appropriate infrastructure to support the implementation and maintenance of these catalogues in each relevant data holder and make them accessible—in a controlled way—to produce secondary datasets.

### 4.3 Automation potential in data curation should be further explored

The data interoperability issues described in Section 3.1. are well known. The innovation in AIDAVA lies in automating a holistic treatment of all these interoperability issues by means of complementary workflows. One data source may present several data interoperability issues, requiring several workflows. Each workflow may include one or more curation tools as well as requests for human intervention when an issue cannot be solved by the machine. Automation in AIDAVA consists of orchestrating the appropriate workflow for each data source and across data sources, to generate a harmonised PHKG from heterogeneous, multimodal data.

With the emergence of powerful new AI tools, such a Large Language Models (LLM) (22), Neuro-Symbolic AI (41), Generalist Medical AI (42) and Medical Imaging, we can expect more and better tools to be available to support the curation of multimodal data.

**Recommendation 3**. Formally describe all potential health data interoperability issues that can occur in health data and define a related data curation workflow with description of needed curation tools and human intervention.

**Recommendation 4**. Maintain a library of data curation tools that can solve the different health data interoperability issues. The library should include an assessment of the tools as well as a formal description of the API, supporting integration.

### 4.4 Data exchange standards are needed but not sufficient: we need a data sharing standard

Source data in health will remain heterogeneous for the foreseeable future. Different formats are in use and/or will soon be mandated: (i) WHO international classification such as ICD required for billing and epidemiological reporting; (ii) the European electronic health record exchange format (EEHRxF) to be mandated by EHDS to support exchange of personal health data based on HL7 FHIR, SNOMED and LOINC already in place in several European countries; (iii) CDISC supporting data collection in the context of drug related regulatory approval; (iv) OMOP typically used as a target format for secondary datasets in clinical research; and (v) many other—often proprietary—formats exist in research and policymaking databases.

Currently, data sources are mapped directly to the required target output, representing n∗m mappings, where n is the number of source formats and m is the number of target formats. This represents a major burden across health and hampers patient care and research. We therefore argue that data exchange standards are needed but not sufficient.

Another possibility is to agree on a data sharing standard, enabling information to be transformed to and from any standard and supporting multiple, but yet unknown, data exchanges; this approach would decrease the number of mappings to n+m. This is the objective of the patient Personal Health Knowledge Graph (PHKG) constrained by the concepts defined in the AIDAVA reference ontology, described in Section 3.2. Although the maintenance of such an ontology is beyond the scope of this project, our aim is to demonstrate the value of an interoperable PHKG for multiple types of exchanges and secondary data use, and to identify guidelines to support the development and maintenance of a global reference ontology encompassing all data exchange standards.

**Recommendation 5**. Develop and maintain an EU-wide (or broader) ontology as the basis for interoperable PHKGs, which supports transformation to main data exchange standards in use (at least EEHRxF and those in use in clinical research such as CDISC, OMOP…)

- Confirm the requirements.
- Review existing/past initiatives (e.g., SNOMED ontological framework, SALUS…) and emerging initiatives (e.g., Precise4Q, EUCAIM Hyper ontology, SPHN…) and develop the European wide foundation layer of the ontology.
- Define and implement a governance process.

### 4.5 Data sharing requires an assessment of the quality of data

Reusing poor quality data has limited value. When developing the requirements for the AIDAVA curation virtual assistant, data users repeatedly asked the same question: how reliable the data are. The answer differs depending on the state of the data: (i) for data sources, a quality label can be established based on the quality level provided by the data holder—if available—including the credentials of the persons who created and validated the data; (ii) for the curated data (i.e., the PHKG), the quality label will be linked to the quality from the source, the level of quality and certification of the curation tools used during transformation, the level of health and literacy of the humans who provided answers when there were semantic gaps, and the number of data quality checks that could not be resolved; (iii) for published data, the quality label will be linked to the level of the curated data, the compliance with the target format, the completeness of the content, the absence of bias as well as the quality, reliability and certification of the imputation algorithm, if applicable.

Article 23.3 (c) of the EHDS mandates to include a data quality statement, such as the completeness and accuracy of electronic health data. Section 5 on health data quality describes the requirements for the quality and utility label for secondary datasets; these requirements map with the question raised by the AIDAVA data users for curated and published data with two major differences: (i) the EHDS requirements include access constraints not addressed in AIDAVA; (ii) the EHDS merges the concept of curated and published data as it only addresses population datasets. In a patient-centric EHDS, one must distinguish the curated PHKG at patient level, and the published output which can be at patient level (e.g., IPS) or at population level (e.g., clinical registry).

**Recommendation 6**. Expanding on Article 23.3 (c) and Article 56, and existing data quality frameworks (43) develop and deploy a quality label framework for each state of data: (i) data sources, (ii) curated data and (iii) published data, with appropriate parameters related to the transformation.

### 4.6 Health data intermediaries, supported by community curators, are needed

The Data Governance Act regulates the setup and functioning of data intermediation services organisations, or what AIDAVA calls ‘health data intermediaries’ (HDI) when they manage health data on behalf of the patient. To our knowledge the most advanced business model of HDI has been developed in the Netherlands through ‘Persoonlijke gezondheidsomgeving’ or Personal Health Environment (44). Such models and organisations, close to the patients, must be further defined and deployed, in alignment with the EHDS regulation, to develop and maintain trust with patients.

To support the patient and their treating physicians, HDIs must equip their customer patients with the appropriate tools to exercise control, agency, and guardianship. This includes a Digital Wallet (45) supporting identity management and linking, dynamic consent management, and data transfer. An AIDAVA-like tool, supported by a catalogue of data sources, will increase the value of data intermediation services by improving the quality of the source data and its value for secondary use, making it a key player in the growing telehealth market, and fostering a genuine health data culture throughout society.

The assumption in AIDAVA is that the automation process will be seamless with maximum automation and minimum of human intervention. When human input is required, it is expected that the patient will be the first person requested to support. The percentage of citizens that will be willing and able to contribute is directly linked to the complexity of the task and will be assessed as part of the prototype evaluation. If we assume that between 5 and 15% of the population will be able to contribute, this means that we need additional support from ‘community curators’, i.e., persons in the community with a minimum or health and digital literacy that would be specifically trained as expert curators and would offer their services to patients through an HDI. Community curators could be a member of the family that would curate the data of the whole family—parents, siblings and children—for free, or could be a third party who should be rewarded for the work done.

It could be argued that this could increase the gap between patients of high and low socio-economic status. While this risk is always present, different approaches should be explored to fund the community curator and data intermediaries (46). There could be a lump sum per patient and per type of diagnosis from national health funding programmes, as high-quality data should reduce the total cost of illness and the cost of research and policy development. There could also be funding from pharmaceutical companies directly to the patient and their community curator, as the availability of interoperable PHKGs could dramatically decrease the cost of trials — as data would be more readily available, just on time — and reduce the decline in the return on investment for research and development (47).

**Recommendation 7**. Define and support deployment development of different models of Health Data Intermediaries to ensure patients can be in control of their data, exercise agency and secure guardianship through an actor close to the patient and chosen by him/her. This includes new organisation models or integration of supporting digital solutions, including digital wallet for the patient as well as maintenance of a catalogue of data sources and data curation services to maintain each individual PHKG within the patient digital wallet.

**Recommendation 8**. Define and pilot the role of community curators, aligned with the Skills data space (48).

## 5 Conclusion

We argue that a patient-centric EHDS will serve foremost each individual patient, but also the population as a whole and other health stakeholders such as healthcare providers and health researchers and policymakers. This mandates the development and maintenance of a high-quality, personal longitudinal health record for each patient, resulting from the curation of their data scattered across multiple systems and organisations. This longitudinal record should be formalised in a Personal Health Knowledge Graph (PHKG) which should be interoperable because it is constrained by a reference ontology; the PHKG should also include a data quality label, derived from the quality of the sources data and the transformations that took place during the curation process.

The AIDAVA project implements a combination of AI-based automation and a ‘human-in-the-loop’ approach, harnessing advanced technologies, human expertise, skill sets, and contextual knowledge to help patients—or their delegates—manage their own data and develop their interoperable, high-quality PHKG. In doing so, patients benefit personally and contribute to the just-in-time production of disposable secondary datasets that promotes research and policymaking. AIDAVA therefore proposes a model that places the patient at the centre of a greener interconnected ecosystem of primary and secondary data use, increasing value for all and for the planet.

Several obstacles need to be overcome to achieve the AIDAVA vision. The first is access to personal health data, not because of data privacy issues, but because of the lack of detailed documentation—including format, data typing, and value restriction—on source data. The definition and enforcement of a catalogue of primary data source should be introduced in EHDS and implemented as a priority. Another important component for the sustainability of AIDAVA-like solutions is the availability of a governed reference ontology, laying the foundations for a global data sharing standard. Additionally, sustainable models for health data intermediaries and supporting community curators need to be defined.

The preliminary results of the AIDAVA project demonstrate that the implementation of a patient-centred EHDS is achievable and beneficial. It requires that the recommendations outlined in this paper are included in the implementing acts being drawn up as part of the EHDS deployment.

## Data availability statement

The original contributions presented in the study are included in the article, further inquiries can be directed to the corresponding authors.

## Author contributions

IZ: Conceptualization, Funding acquisition, Investigation, Methodology, Project administration, Writing – original draft. KN: Investigation, Writing – review & editing, Methodology. DS: Conceptualization, Funding acquisition, Methodology, Writing – review & editing. HM: Conceptualization, Funding acquisition, Methodology, Writing – review & editing. DK: Conceptualization, Funding acquisition, Methodology, Writing – review & editing. BS: Investigation, Writing – review & editing. IC: Writing – review & editing. SS: Conceptualization, Funding acquisition, Methodology, Writing – review & editing. KU: Writing – review & editing. PK: Investigation, Writing – review & editing. E-ML: Writing – review & editing, Conceptualization, Funding acquisition, Investigation. MB: Writing – review & editing. MD: Conceptualization, Funding acquisition, Investigation, Methodology, Writing – review & editing, Supervision. RC: Conceptualization, Funding acquisition, Investigation, Methodology, Project administration, Writing – review & editing.

## Footnotes

^1^Privacy Preserving Record Linkage obfuscating or encrypting Personal Identification Information supports record linkage; however, by its nature, it masks personal identifiable information.

^2^Inclusion of clinical research data has been requested by the European Parliament in their comments from November 2023.

^3^It is expected that this will be included in the EEHRxF format.

^4^If concepts are not available, when onboarding data sources into the system, they are added to the reference ontology following the defined governance process.

^5^Presentation during the HealthData@EU Pilot—Forum on October 19th.
