# Erratum: Artificial intelligence based data curation: enabling a patient-centric European health data space

**DOI:** 10.3389/fmed.2024.1455319

**Published:** 2024-07-09

**Authors:** Frontiers Production Office

**Affiliations:** Frontiers Media SA, Lausanne, Switzerland

**Keywords:** AI-based data curation, personal health knowledge graph, ontology, catalogue of data sources, EHDS, data intermediary, patient-centricity

Due to a production error, two author names were incorrectly spelled as “Eno-Martin Lotmam” and “Stefan Shulz.” The correct spelling is “Eno-Martin Lotman” and “Stefan Schulz” respectively.

The publisher apologizes for this mistake. The original article has been updated.

